# Expanded dengue syndrome presented with rhabdomyolysis, compartment syndrome, and acute kidney injury: A case report

**DOI:** 10.1097/MD.0000000000028865

**Published:** 2022-02-18

**Authors:** Aabiya Arif, Muhammad Raheel Abdul Razzaque, Lucas Marian Kogut, Sameer Saleem Tebha, Filza Shahid, Mohammad Yasir Essar

**Affiliations:** a Department of Medicine, Ziauddin University, Karachi, Pakistan; b Department of Medicine, Section of Nephrology, Jinnah Medical and Dental College, Karachi, Pakistan; c Department of Nephrology, Hope Medical Institute, Virginia, United States of America; d Department of Neurosurgery and Neurology, Jinnah Medical and Dental College, Karachi, Pakistan; e Department of Medicine, Jinnah Medical and Dental College, Karachi, Pakistan; f Kabul University of Medical Sciences, Kabul, Afghanistan.

**Keywords:** acute kidney injury, case report, compartment syndrome, expanded dengue syndrome, rhabdomyolysis

## Abstract

**Rationale:**

Expanded dengue syndrome (EDS) is the phenomenon coined by the World Health Organization for cases of dengue fever (DF) with rare but dangerous consequences. EDS mainly leads to complications involving the vital organs, thus is also associated with a higher mortality rate. This case report presents the first-ever case where a patient developed EDS with both rhabdomyolysis-induced acute kidney injury and compartment syndrome of the limbs.

**Patient concerns:**

Our patient, an 18-year-old Pakistani male, presented with fever, colicky abdominal pain, vomiting, diarrhea, dark-colored urine, and oliguria.

**Diagnoses:**

Dengue rapid NS-1 test came back positive. Along with myoglobinuria both serum creatine phosphokinase and creatine levels were abnormal. Hence, the patient was diagnosed with rhabdomyolysis-induced acute kidney injury. On physical examination, his right arm was painful and tender with restricted movement at the elbow. A Doppler ultrasound of the arm revealed thickening of the skin and underlying muscles, as well as edematous abnormalities affecting the entire right upper limb, both of which are indications of compartment syndrome.

**Interventions and outcome:**

The management included rehydration, administration of dextrose and bicarbonate (bicarbonate infusion) prepared by adding 150 mEq sodium bicarbonate in 850 mL dextrose 5%, pain killers, along with platelet, and packed red cell transfusions. Additionally, right upper limb was kept elevated at 90° for 30 minutes every 2 hours to reduce edema and crept bandages were applied. The patient was discharged after 11 days and the follow-up was uneventful.

**Lesson:**

Physicians should be aware that rhabdomyolysis-induced acute kidney damage and limb compartment syndrome are also possible DF consequences, and they should be on the lookout for any indications pointing to these complications in DF. A prompt diagnosis can prevent further complications and fatality.

## 1 Introduction

Dengue fever (DF) is a mosquito-borne tropical disease caused by female Aedes aegypti. Belonging to the Flavivirus family, it has 4 unique serotypes (DEN1–4). The incubation period is 7 to 10 days. It is currently endemic in 128 countries, mostly developing countries, putting an estimated 3.97 billion population at risk each year.^[^1^,^^2^^]^ Clinical illness can range from asymptomatic DF to life-threatening dengue hemorrhagic fever (DHF) and dengue shock syndrome (DSS). DHF and DSS are related to worse outcomes in dengue patients. These severe clinical states manifest as a multiorgan failure involving the heart, brain, liver, lungs, and kidneys.

DF is mostly a self-limiting disease with comparatively low mortality, characterized by fever with chills, myalgias, arthralgia, headache, retro-orbital pain, vomiting, and rash. However, there are many other rare yet serious manifestations that are observed in patients with DF which are labeled as expanded dengue syndrome (EDS) and its serious manifestations are labelled as DHF and DSS as mentioned above.

EDS mostly causes damage to the liver, kidney, heart, brain, or bone marrow. Rhabdomyolysis-induced acute kidney injury is one of the renal manifestations of EDS, which is characterized by elevated serum creatine kinase and increased excretion of myoglobin in the urine along with an increase in serum creatine.^[^2^,^^3^^]^

Compartment syndrome, a painful condition due to increased intra-compartmental pressure that impairs circulation and renders the limb at risk of ischemia due to pressure related decreased arterial blood flow, pressure induced nerve damage that causes pain, and cause decreased venous blood flow that leads to edema^[4]^ Compartment syndrome of the limbs is one of the rarest complications in DF, with just 2 reported cases in current literature. We present the first-ever case of a young man from Pakistan who developed both rhabdomyolysis induced acute kidney injury (AKI) and compartment syndrome in the right arm concomitantly, secondary to dengue.

## 2 Case presentation

An 18-year-old, Pakistani, previously healthy male with no known comorbidities, presented to the emergency room with a history of high grade, intermittent fever with chills and rigor for the past 7 days, generalized colicky abdominal pain, vomiting, and dark-colored urine for 5 days. He also complained of foul-smelling, non-bloody non-mucoid diarrhea for 3 days, and no urine output for the previous 18 hours. He also complained of headaches, vertigo, generalized weakness, and myalgia with severe pain in his right arm.

There was no history of retro-orbital pain, neck stiffness, photophobia, and no other urinary symptoms.

On physical examination, he was pale and dehydrated, his cardiovascular, respiratory, abdominal, and central nervous systems were all normal. Musculoskeletal and skin examination revealed extremely tender right arm, it was edematous, bruises, limited active and passive movements at the elbow joint due to pain. In comparison to the other arm, the overlying skin was warm, and there were few bullae visible, with blood and fluid leaking out.

Upon admission, his vitals were as follows: blood pressure – 112/72 mm Hg, pulse – 120 beats/min, respiratory rate – 26 breaths/min, oxygen saturation – 96% on room air.

Laboratory investigation revealed thrombocytopenia and increased levels of serum total bilirubin, direct bilirubin, creatine, urea, alanine aminotransferase/serum glutamic pyruvic transaminase, aspartate aminotransferase/serum glutamic oxaloacetic transaminase, and creatine phosphokinase. The investigations are mentioned in Table 1.

**Table 1:** Summary of laboratory investigations.

Investigations	Day 1	Day 2	Day 3	Day 4	Reference value
Hemoglobin (g/dL)	14.10	10.70	6.20	8.40	13.7–16.3
Total WBC (count/L)	7.22	12.23	26.11	16.14	4.0–11.0
Platelets (10^9^/L)	14	47	56	80	150–240
Hematocrit level (volume/%)	29.1	38.3	17.8	24.8	41.9–48.7
Creatine (mg/dL)	2.47	1.76	1.38	1.29	0.9–1.3
Creatine phosphokinase (U/L)	5232	4061	–	–	46–171
Urea (mg/dL)	57	59	52	49	10–50
Alanine transaminase (U/L)	380	–	252	235	<45
Aspartate aminotransferase (U/L)	837	–	626	631	<31
Total bilirubin (mg/dL)	1.31	1.31	–	1.07	<1.2
Direct bilirubin (mg/dL)	0.65	0.87	–	0.59	<0.2

WBC = white blood cells

Dengue rapid NS-1 test was positive. Serologic tests for malaria, typhoid, hepatitis B & C were negative. The urine and blood cultures were also sent at the time of initial workup and they came “no growth” after 3 and 5 days, respectively.

**Figure 1:**
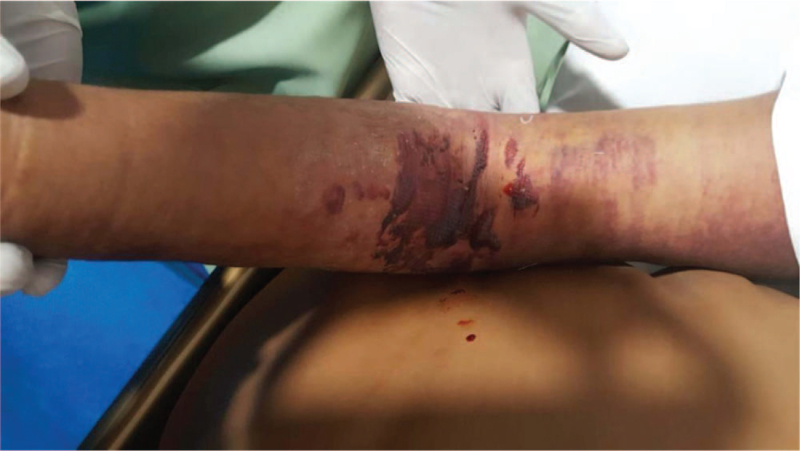
Blister site with anechoic zones demonstrative of compartment syndrome.

An ultrasound Doppler scan of the right arm was ordered, that indicated thickening of the skin and underlying muscles, as well as edematous abnormalities affecting the entire right upper limb. There were no signs of any pockets of collection. Blister sites were found to have anechoic zones as shown in Figure 1.

Abdominal ultrasound showed altered texture liver and streak of fluid seen around the liver that was minimal. Bilateral normal sized kidneys. Rest of the structures, such as spleen, gall bladder, and urinary bladder were all unremarkable. There was no significant past medical, surgical, psychosocial, and family history. The patient management is summarized in Table 2.

**Table 2:** Management of the patient is summarized.

Name	Dosage	Route of administration
Normal saline	75 mg/kg on flow	IV
Nurobion	1 ampule once daily	Injection
Tramadol	2 mg/h on flow	IV
Famotidine	20 mg/12 hourly	Peroral
Multibionca (multivitamins)	1 ampule once daily	IV
Mucolator sachet (acetyl cysteine)	200 mg 8 hourly	Peroral
Dextrose + bicarbonate	100 mL/h on flow	IV
Paracetamol	1 g/100 mL 8 hourly	IV
Gravinate	8 hourly	IV
Lexotinil	3 mg	Peroral
Beneprotien	150 mL	Peroral
Ensure milk	150 mL	Peroral

IV = intravenous.

The patient was also given 1 platelet transfusion and 3 packet cell transfusions to support treatment and minimize consequences of thrombocytopenia secondary to dengue, as he developed large ecchymosis patches over right arm and forearm and for his anemia, respectively. In addition to this, the arm was kept elevated at 90° for 30 minutes every 2 to 3 hours to reduce edema and crepe bandage was applied to the right arm, the dressing was changed twice daily. The patient was discharged after 11 days, and follow-up was uneventful.

## 3 Discussion

DF, DHF, and DSS are all names given to different symptomatically distinct dengue manifestations. The World Health Organization, in 2012, coined the term “expanded dengue syndrome” to describe patients that do not fit into either DHF or DSS but show atypical symptoms in vital organs systems such as the cardiovascular system, neurological system, kidneys, gut, and hematological system. EDS is now being increasingly used in literature around the world, as it encompasses the rare atypical and uncommon symptoms of dengue, which we are seeing in recent times since the severity and spectrum of disease in DF has broadened.^[^2^,^^5^^]^

To our knowledge, this is the first case of EDS where a patient presented simultaneously with compartment syndrome of the right upper limb with laboratory workup showed rhabdomyolysis-induced AKI. However, rhabdomyolysis-induced AKI and compartment syndrome of the upper limb, in patients with DF have been documented separately a few times. In the existing literature, there are 2 case reports that presented with compartment syndrome of the upper limb and only 7 case reports of rhabdomyolysis-induced AKI in DF were identified (Table 3). Myalgia and oliguria were noted in all 8 studies, dark urine in 5, and renal replacement therapy was required in 5 patients. In total, there were 7 recoveries, with the outcome not recorded by Acharya et al.

**Table 3:** Summary of the existing literature on rhabdomyolysis induced acute kidney injury in dengue fever.

Author, year, country	Gender, age	Type of dengue	Myalgia	Muscle weakness	Dark urine	Urinary myoglobin	Oliguria	Serum creatine phosphokinase (IU/L)	Serum creatine(mg/dL)	Renal biopsy	Renal replacement therapy	Outcome
Gunasekera et al, 2000, Ceylon^[12]^	Female, 28 yrs	NR	Yes	Yes	Yes	Yes	Yes	>5000	8.8	No	PD, HF	Recovery
Acharya et al, 2010, India^[13]^	Male, 40 yrs	NR	Yes	NR	Yes	Yes	Yes	29,000	2.6	No	NR	NR
Repizo et al, 2013, Brazil^[14]^	Male, 28 yrs	DF	Yes	Yes	NR	ND	Yes	4063	11.6	ATN	HD	Recovery
Aruna Wijeshinghe et al, 2013, Sri Lanka^[15]^	Male, 42 yrs	DF	Yes	NR	NR	Yes	Yes	6240	6.3	No	HD	Recovery
Jha et al, 2013^[16]^	Male, 21 yrs	DF	Yes	NR	Yes	NR	Yes	71,500	10.2	No	HD	Recovery
Mishra et al, 2015^[17]^	Male, 21 yrs	DF	Yes	NR	Yes	NR	Yes	7800	2.7	No	ND	Recovery
Tansir et al, 2017, India^[5]^	Male, 35 yrs	DF	Yes	NR	NR	ND	Yes	6400	9.5	No	HD	Recovery
Current case, 2021, Pakistan	Male, 17 yrs	DF	Yes	Yes	Yes	ND	Yes	5232	2.47	No	ND	Recovery

ATN = acute tubular necrosis, DF = dengue fever, HD = hemodialysis, HF = hemofiltration, ND = not done, PD = peritoneal dialysis, NR = not reported.

Dengue-associated rhabdomyolysis is characterized by myalgia, black urine, and substantially increased CPK values. These symptoms arise due to muscle necrosis leading to the release of muscle enzymes like creatine phosphokinase, lactate dehydrogenase, aldolase, alanine aminotransferase, aspartate aminotransferase, and myoglobin in the systemic circulation.^[6]^

The pathophysiology behind the onset of rhabdomyolysis in DF patients remain unknown.^[7]^ However, rhabdomyolysis has been reported in a variety of viral illnesses, including influenza A and B, coxsackievirus, Epstein-Barr virus, and HIV.^[8]^ Thus, it is not surprising that the dengue virus could cause rhabdomyolysis because it shares several traits with other viruses that cause severe myositis and rhabdomyolysis.^[7]^

Acute kidney injury is a life-threatening complication. AKI occurs in 33% to 50% of rhabdomyolysis patients and is associated with severe renal failure.^[6]^ Acute renal damage with myoglobinuria is the most serious consequence of both traumatic and non-traumatic rhabdomyolysis.^[8]^ Although the precise mechanisms through which rhabdomyolysis impairs renal function are uncertain, experimental studies have suggested possible pathways, which include: Damaged myocytes sequester fluid, resulting in hypovolemia, which activates the renin-angiotensin system, resulting in renal vasoconstriction. Myoglobin, in particular, has a direct toxic effect on renal tubules, particularly the proximal tubule. In the presence of acidic urine, excess myoglobin can interact with Tamm-Horsfall protein in the distal tubules, resulting in cast formation leading to tubular blockage.^[9]^

Although rhabdomyolysis is a well-known risk factor for AKI, other concurrent damage factors such as hypovolemia or dehydration, and acidosis may also need to be present in order for clinically meaningful myoglobinuria-induced renal injury to occur.^[^6^,^^12^^]^

Our patient on arrival was dehydrated in addition to DF and rhabdomyolysis, which lead to the development of AKI with a CPK level of more than 5000. Clinically, there was compartment syndrome of the right arm, board like rigidity and severe tenderness. The most prevalent causes of rhabdomyolysis are traumatic, commonly seen are tibial or forearm fractures, bleeding, crush injuries, or prolonged limb compression, such as plaster casts.^[10]^

There are currently only 2 case reports that describe upper-limb compartment syndrome because of dengue infection. In DF, there are 2 mechanisms that lead to the development of compartment syndrome. In a case report provided by Khoo et al,^[10]^ a hematoma at the site of a prior arterial line caused compartment syndrome in the right arm due to thrombocytopenia (11.2 g/dL and 30 × 109/L).

In a case report described by Bandyopadhyay et al,^[11]^ systemic capillary hyperpermeability produced by the production of a spectrum of cytokines in DF caused compartment syndrome in the right forearm. An increase in vascular permeability leads fluid to escape from the intravascular to the interstitial compartment, resulting in capillary leak syndrome.

The latter mechanism is thought to be the reason for the development of compartment syndrome in our patient as the ultrasound Doppler revealed tissue edema that was seen in the forearm and no hematoma as documented in the first case. The presence of multiple prick marks of his forearm and arm for possible collection of blood samples, mal positioned cannula for managing fluids and sending samples for arterial blood gases may have caused excessive fluid leak in muscle planes, raised local pressure and at its extreme caused muscle damage that gives us a combined manifestation of compartment syndrome, rhabdomyolysis, and acute kidney injury in our patient.

## 4 Conclusion

Due to the rise in cases with rhabdomyolysis-induced AKI in patients with DF, a close eye on patient's urine output and color, evaluating urinary complaints on history and sending CPK levels should be done in patients with DF for early diagnosis and management. Moreover, any similar case reports need to be documented and persuade for publication in literature so that healthcare workers have a better insight and familiarity dealing with such rare complications of DF.

## Author contributions

**Conceptualization:** Filza Shahid.

**Writing – original draft:** Aabiya Arif, Muhammad Raheel Abdul Razzaque, Lucas Marian Kogut, Sameer Saleem Tebha, Filza Shahid.

**Writing – review & editing:** Lucas Marian Kogut, Mohammad Yasir Essar.
